# Efficacy of glucocorticoids treatment in recurrent embryo implantation failure

**DOI:** 10.1038/s41420-025-02753-w

**Published:** 2025-10-16

**Authors:** Qin-Yu Cai, Wei-Zhen Tang, Zhi-Mou Li, Jia-Zheng Li, Xing-Qi Zhi, Qin-Hao Yang, Jie Sheng, Tai-Hang Liu

**Affiliations:** 1 Department of Bioinformatics, School of Basic Medical Sciences, Chongqing Medical University, Chongqing, China; 2 The Joint International Research Laboratory of Reproduction and Development, Chongqing Medical University, Chongqing, China; 3 School of Life Sciences, Westlake University, Hangzhou, China; 4 Department of Pathology, China-Japan Friendship Hospital, Beijing, China

**Keywords:** Autoimmunity, Reproductive disorders, Immunopathogenesis, Drug development, Translational immunology

## Abstract

Recurrent implantation failure (RIF) poses a significant challenge in treating infertility. RIF can stem from various factors, commonly involving immune issues. Recent literature suggests that glucocorticoids (GCs) can effectively inhibit immune responses in RIF patients. However, a recent multicenter randomized controlled trial found that oral prednisone did not enhance live birth rates in RIF cases. Despite their potential benefits, the diversity in dosage regimens and treatment protocols highlights the need for further research to establish standardized guidelines and ensure long-term safety. This review explores the use of GCs treatments for RIF, including their use alone and in combination with other medications. It emphasizes the necessity for solid evidence to determine the effectiveness and safety of these treatments. Future studies should focus on defining optimal dosages, treatment durations, and administration methods tailored to different RIF patient profiles. Moreover, well-designed randomized trials are crucial to assess the efficacy and risks associated with GCs in treating RIF.

## Facts

- GCs can effectively suppress inflammatory and immune reactions in the endometrial lining, while they are potent immunomodulators that have been used worldwide to improve embryo implantation and prevent miscarriage.
- Recent large multicenter, randomized, double-blind, placebo-controlled study demonstrated that oral prednisone, a GCs drug, did not improve the live birth rate in RIF patients which challenged the clinic guidelines in numerous countries.
- Using GCs in RIF treatments in clinic may have varying effects and side effects owing to different types, dosages, routes, and timings of glucocorticoids as well as the combination used GCs with low-dose aspirin and low-molecular-weight heparin.
- Clinical guidelines in a mass of countries directly or indirectly recommend the use of GCs for RIF have been challenged, highlighting the need for the more high-quality research to determine appropriate dosages, durations, and administration methods.

## Introduction

Human embryo implantation is a highly demanding and complex process, consisting of embryo apposition, adhesion, penetration, and trophoblast invasion. Implantation is considered successful when an intrauterine gestational sac is detected via ultrasound scan. Failure at any of these steps can result in implantation failure [1]. It’s well acknowledged that implantation failure is closely related to various benign gynecological diseases, such as endometriosis, hydrosalpinx, leiomyoma polycystic ovary syndrome, etc [2]. Despite advances that have been made in drug treatment, surgical removal of lesions, and hormonal therapy, up to one in six couples still suffer from infertility issues [3]. Assisted reproductive technology (ART) has been increasingly utilized to help infertile couples improve their chances of conception, yet recurrent implantation failure (RIF) remains a perplexing issue [4]. RIF has exacerbated the psychological burden and pain for at least 10% of patients undergoing in vitro fertilization-embryo transfer worldwide, drawing widespread attention to its pathogenesis and treatment methods [5].

RIF refers to a condition in which multiple attempts at embryo transfer during in vitro fertilization (IVF) or ART cycles fail to result in successful implantation and establishment of pregnancy. However, no universally agreed upon diagnostic criteria for RIF currently exist owing to mainly lack of consensus manifests in two key aspects: variable diagnostic criteria and heterogeneous inclusion parameters. Several factors might be involved in the pathogenesis of RIF, including underlying problems with the gametes, the embryos, and endometrial receptivity. The immune system plays a central role in establishing receptivity and initiating pregnancy, having unique effects in modulating the decidual response, epithelial-embryo attachment, trophoblast invasion, vascular adaptation, immune tolerance, and cytokine balance [6]. The role of the immune system in RIF has gained significant attention, particularly changes in cell-mediated immunity characterized by heightened cytotoxicity and excessive inflammatory stimuli, prompting a primary focus on T cells and NK cells in most studies. Since the first discovery of uterine NK (uNK) cells in 1991, most evidence supports the importance of uNK cells during pregnancy [7]. However, recent studies have highlighted that uNK might also contribute to adverse pregnancy outcomes by inducing autoimmune diseases and other mechanisms [8].

Glucocorticoids (GCs) are a class of steroid hormones with potent anti-inflammatory and immunosuppressive properties. GCs can effectively suppress inflammatory and immune reactions in the endometrial lining, while they are potent immunomodulators that have been used worldwide for many years to improve embryo implantation and prevent miscarriage [9, 10]. Numerous studies have revealed that it promotes the proliferation and invasion of trophoblast cells, normalizes the expression of intrauterine cytokines and the activity of natural killer cells, and stimulates the secretion of human chorionic gonadotropin (hCG) [11]. GCs are inexpensive, convenient, and generally considered safe at low doses in clinics compared to other immunomodulatory therapies [12]. Therefore, clinical guidelines in a mass of countries directly or indirectly recommend the use of GCs for RIF [13]. A few retrospective and prospective studies have reported that GCs can have a positive impact on implantation rates and clinical pregnancy success rates of the women. Nevertheless, all of these studies had the limitations of small sample sizes. Currently, a recent large multicenter, randomized, double-blind, placebo-controlled study demonstrated that oral prednisone, a GCs drug, did not improve the live birth rate in RIF patients [14]. This has once again pushed the GCs treatment for RIF patients to the spotlight of debate. However, these studies were limited by methodological weaknesses, such as combined regimens with either low-dose aspirin (LDA) or low-molecular-weight heparin (LMWH), varying definitions of RIF across different ART centers, and different dosages. Further studies are required to determine whether the use of GCs can be considered an effective treatment method.

Despite the clinical relevance of concurrent GCs and RIF treatment reporting - particularly given their global therapeutic implications for millions of RIF patients - persisting knowledge gaps regarding GCs efficacy and the absence of evidence-based treatment guidelines continue to greatly hinder clinical decision-making. This review delves into the pharmacological mechanisms and efficacy of GCs therapy in addressing RIF, whether to use GCs alone or combined regimens of LDA, LMWH, and other drugs. Furthermore, this review aims to provide a critical analysis that lays the foundation for future research to establish robust hypotheses and effective treatment protocols.

## Definition of RIF

The inconsistent definition of RIF arises from the variations in variable diagnostic criteria and heterogeneous inclusion parameters with RIF women, such as research type, the racial makeup of the population, and geographic location, leading to different reports on its incidence rate. These discrepancies are among the primary reasons why the epidemiological status of RIF has not yet been comprehensively examined. Currently, the widely accepted definition of RIF, as suggested by Simon and Laufer in 2012, involves undergoing three or more embryo transfer cycles (comprising both fresh and frozen transfer cycles). During each cycle, 1 or 2 high-quality embryos are implanted, yet clinical pregnancy remains unattained [15]. In 2014, Coughlan et al. proposed that for patients under 40 years old undergoing IVF-ET, the definition of RIF should entail at least 4 high-quality embryos transferred during a minimum of 3 fresh or frozen cycles, still without achieving clinical pregnancy [16]. As of 2023, the newly issued consensus from Chinese experts on the clinical diagnosis and treatment of repeated implantation failure suggests defining RIF as the failure to achieve clinical pregnancy in women under the age of 40 after transferring at least 3 high-quality embryos within 3 fresh or frozen cycles. This includes both day 3 embryos (with a cell count of ≥ 8, uniform blastomeres, and a fragment rate of <10%) and blastocysts (≥ 3BB) [17]. It’s mean that RIF is defined as failure to achieve pregnancy after a variable number of attempts based on patient’s age and use of euploid or untested embryos if the expected cumulative probability of achieving pregnancy was exceeding a cut-off of 60% and no pregnancy achieved yet.

Currently, there is no uniform standard for the diagnosis and treatment of RIF, and different centers often adjust their definitions to suit their own needs in clinical practice [18]. A significant issue is the frequent oversight of many crucial factors that impact the success of implantation, encompassing embryonic, maternal, and paternal factors [19]. Some of these factors are routinely screened for, while others are not, but they may be considered for screening if a diagnosis of RIF is made. Hence, in recent times, authors have proposed personalized definitions for implantation failure, such as embryo aneuploidy (and consequently, oocyte age) is arguably the most critical factor contributing to the failure of ART factors in anticipated blastocyst euploidy rates across categories of female age, euploid blastocyst implantation rate, and a specified threshold of cumulative probability of implantation. Although RIF does not have a universal definition, numerous research and clinical practices provide information to draw a practical definition of RIF. To address ambiguities in the definition to date, the ESHRE PGD Consortium recommended that RIF refers to a situation in which the transfer of embryos deemed viable has repeatedly failed to yield a positive pregnancy test in a specific patient, prompting the need for further investigations and potential interventions in 2023 [20]. This definition adopts an individualized approach, which doesn’t rely on a ‘one size fits all’ criterion (such as a fixed number of embryos transferred). Instead, it takes into consideration, at least in part, the factors known to influence an individual patient’s likelihood of conception. We suggest employing extensive datasets and advanced algorithms to achieve personalized diagnosis by modeling multiple factors and further defining RIF in the future.

## The Immunological Pathology of RIF

### Immune cells

#### Uterine Nature Killer cells

**Fig. 1:**
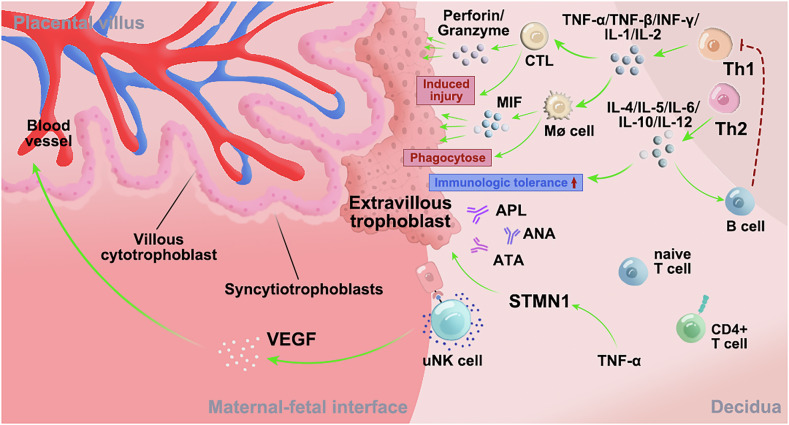
The main involvement of immune cells and immune mechanism of recurrent implantation failure at the Maternal-fetal interface. APL Antiphospholipid Antibodies, ANA Anti-nuclear Antibody, ATAs Anti-thyroid Antibody, UNK cell Uterine Natural Killer Cell, TH1 T Helper Type 1 cell, TH2 T Helper Type 2 cell, CD4 + T cell CD4-positive T cell, Native T cell Native T cell, CTL Cytotoxic T Lymphocyte, MØ cell Macrophage, B cell B Lymphocyte, TNF-α Tumor Necrosis Factor-alpha, TNF-β Tumor Necrosis Factor-beta, IFN-γ Interferon-gamma, IL-1 Interleukin-1, IL-2 Interleukin-2, IL-4 Interleukin-4, IL-5 Interleukin-5, IL-6 Interleukin-6, IL-10 Interleukin-10, IL-12 Interleukin-12, VEGF Vascular Endothelial Growth Factor, MIF Macrophage Migration I.

The main involvement of immune cells and immune mechanism of RIF at the maternal-fetal interface was shown in Fig. 1. Uterine Nature Killer (uNK) cells play a crucial role in RIF. These cells express killer immunoglobulin-like receptors (KIRs), which interact with trophoblast human leukocyte antigen-C (HLA-C) molecules. This interaction regulates embryo invasion. In assisted reproduction, the oocyte’s HLA-C allele differs genetically from that of surrogate mothers, leading to an increased presence of non-self HLA-C2 molecules presented to uNK cell KIR receptors. This triggers an immune inhibitory signal on uNK cells, potentially causing arterial transformation disorders, particularly in women with a KIR AA genotype.

Under pathological conditions associated with RIF, the number of uNK cells increases, and these cytotoxic uNK subsets fail to interact normally with extravillous trophoblasts (EVT), leading to EVT damage due to heightened cytotoxicity and the release of proinflammatory factors like TNF-α, which contribute to necrosis, apoptosis, and inflammation [21]. The overexpression and secretion of these proinflammatory factors also down-regulate the expression of stathmin 1, thereby inhibiting trophoblast cell growth and reproduction. This results in a lack of EVT invasion [22]. uNK cells are major sources of vascular endothelial growth factor (VEGF). Studies have shown both increased and decreased VEGF expression in women with RIF, along with variations in its polymorphisms [23]. These discrepancies may arise from differences in sampling time, sample types, and methodologies. While some research suggests that altered VEGF expression could contribute to excessive oxidative stress and implantation failure, others indicate a protective role. We emphasize the need for further evidence to fully understand the relationship between abnormal uNK cell secretion of VEGF and RIF.

#### Peripheral Nature Killer cells

Peripheral NK cells may exert a potentially damaging effect on in vitro fertilization due to their cytotoxicity, which is mediated by perforin, granzymes, and Fas ligand, as well as through various cytokines such as TNF-α and IFN-γ. Additionally, NK cells play a role in regulating T cell responses, either directly or indirectly [24]. The majority of existing studies suggest that elevated levels of NK cells may contribute to reproductive failure [25]. However, recent research has challenged this notion by proposing that peripheral NK cells do not significantly influence the onset of implantation failure [26].

In this particular study, neither the mean numbers of CD16 + CD56 + NK cells nor the mean numbers of activated CD16 + CD56 + CD69 + NK cells in the RIF groups were significantly different from those in the control group, and no significant correlation was found between the levels of NK cells (whether activated or not) and IFN-γ in the RIF patients.

#### T Cells

T cells are categorized into three primary groups based on their functions: CD4 + T helper (Th) cells, CD8+ cytotoxic T cells, and CD4 + CD25+ regulatory T cells [27]. Among these, Th cells have been the most extensively studied, particularly focusing on subtypes such as Th1, Th2, and Th17 [28]. Current research emphasizes the roles of Th1 and Th2 cells in reproductive processes. A prevailing hypothesis suggests that an elevated Th1-to-Th2 ratio may contribute to implantation failure [29]. Th1 cells are known for producing pro-inflammatory cytokines, including TNF-α, TNF-β, IFN-γ, IL-1, and IL-2. These cytokines activate cytotoxic T cells and macrophages, thereby stimulating cellular immunity and promoting inflammatory responses in the uterus. This inflammation can suppress trophoblast growth, potentially leading to implantation failure.

In contrast, Th2 cells play a role in autoantibody production and protect the fetus against infection by producing anti-inflammatory cytokines such as IL-4, IL-5, IL-6, IL-10, and IL-12. Th2 cells also release cytokines that establish local Th2 dominance, fostering maternal-fetal tolerance [30].

Interestingly, while predominant Th1-type immunity is often observed in cases of abortion, some studies have reported excessive Th2 activity in recurrent pregnancy loss [31]. This discrepancy may arise from the autoantibody production by Th2 cells, which could lead to autoimmune diseases or negatively impact implantation through other mechanisms.

### Autoimmune disorders

#### Antiphospholipid antibodies

Antiphospholipid syndrome (APS) is a systemic autoimmune disorder defined by persistent antiphospholipid antibodies accompanied by either thrombotic events (venous/arterial/microvascular thrombosis) or obstetrical complications (recurrent pregnancy loss, severe preeclampsia, or placental insufficiency). The antiphospholipid antibodies (aPL) can induce a procoagulant state at the maternal-fetal interface, impair trophoblast invasion and differentiation, and trigger complement-mediated inflammatory responses, all of which are implicated in the pathology of RIF [32] with several studies providing evidence of its involvement. In 2019, the European Register of Obstetrical APS (EUROAPS) published findings from a cohort of 1000 obstetric APS patients, revealing a miscarriage prevalence of 38.6%, indicating an association between APS and unfavorable pregnancy outcomes [33]. Additionally, a meta-analysis highlighted a higher prevalence of aPL in women experiencing RIF compared to fertile women, with anticardiolipin-IgG positivity being 5.02 times more common among those with RIF [34].

Antiphospholipid antibodies adversely affect trophoblast function by impairing proliferation, syncytialization, and invasion. Di Simone et al. reported a decrease in the Bcl-2/Bax ratio in trophoblast cells treated with aPL, suggesting a pro-apoptotic effect, although no definitive pro-apoptotic mechanism was identified [35]. Furthermore, aPL induces downregulation of α1 integrin, VE-cadherin, and matrix metalloproteinases while upregulating α5 integrin, E-cadherin, and inhibitors of metalloproteinases [36], suppressing the secretion of hCG from human placental explants, thereby impairing trophoblast invasion disrupt trophoblast cell adhesion molecules, and activate the complement system on the trophoblast surface, thereby inducing a pro-inflammatory response. On the other hand, they induce microvascular thrombosis, disrupting decidualization and resulting in an insufficient endometrial environment for proper embryo invasion, reducing endometrial receptivity during the window of embryo implantation, further contributing to the risk of implantation failure [37].

#### Anti-nuclear antibodies

Anti-nuclear antibodies (ANAs) are associated with systemic autoimmune disorders and have long been recognized for their potential negative impact on IVF. These antibodies contribute to the pathogenesis of various systemic autoimmune conditions, such as systemic lupus erythematosus (SLE), lupus nephritis, and Sjögren’s Syndrome [38]. Such disorders can negatively influence pregnancy outcomes [39]. Additionally, ANAs present in follicular fluid may directly affect embryonic development by promoting trophoblast apoptosis and inhibiting proliferation.

#### Anti-thyroid antibodies

Anti-thyroid antibodies (ATAs), which can contribute to mild thyroid dysfunction and reflect reduced thyroid functional capacity, have been shown to negatively influence pregnancy outcomes [40]. It is well-established that ATAs can cross the blood–ovarian barrier and are detectable in the follicular fluid of women with infertility and thyroid autoimmunity [41, 42]. In an experimental study, Kelkar et al. observed cross-reactivity between anti-zona pellucida antibodies derived from sera of women with autoimmune oophoritis and mouse thyroid tissue [43]. Based on this finding, it has been suggested that anti-thyroid antibodies may bind to the zona pellucida, disrupting early embryonic development processes such as fertilization and hatching. This interference could ultimately result in reduced implantation success. Additionally, ATAs increase the risk of ovarian reserve decline, impairing fertility in women of reproductive age and potentially leading to a higher likelihood of IVF failure.

## The Pharmacological Mechanism and Application Status of Glucocorticoids in RIF

**Fig. 2:**
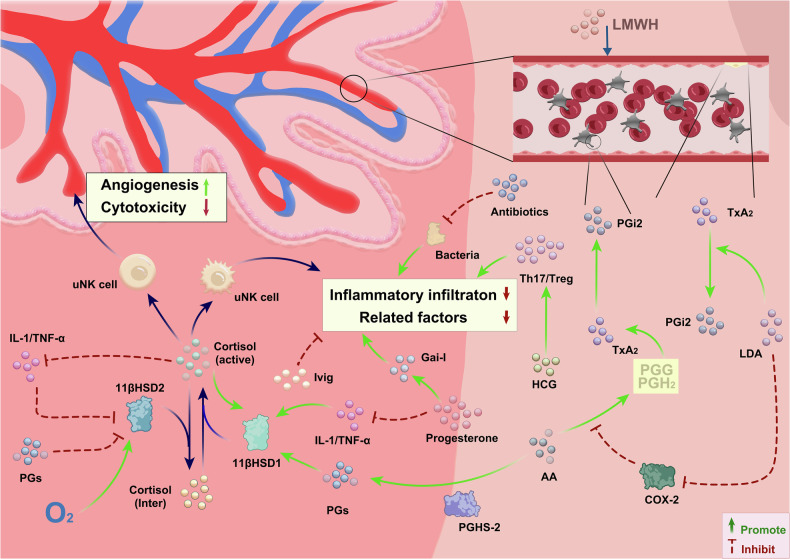
The molecular mechanism of main therapeutic drugs for recurrent implantation failure. UNK cell Uterine Natural Killer Cell, IL-1 Interleukin-1, TNF-α Tumor Necrosis Factor-alpha, AA Arachidonic Acid, PGI2 Prostaglandin I2, TxA2 Thromboxane A2, PGs Prostaglandins, GHS-2 Growth Hormone Secretagogue-2, Gai-I Gi protein, HCG Human Chorionic Gonadotropin, Ivig Intravenous Immunoglobulin, LDA Low-Dose Aspirin, LMWH Low Molecular Weight Heparin.

Figure 2 manifests the molecular mechanism of main therapeutic drugs for RIF. RIF remains a challenging issue in the field of infertility, with extensive and latest data indicating that at least 36 different therapies for RIF patients have been attempted in clinical studies [44]. Immune therapies have emerged as a promising approach in the treatment of RIF, aiming to modulate the immune system to enhance endometrial receptivity and support successful implantation [45, 46]. Current immunomodulatory approaches primarily involve intrauterine infusion of peripheral blood mononuclear cells (PBMCs), intrauterine administration of platelet-rich plasma (PRP), and subcutaneous injection of granulocyte colony-stimulating factor (G-CSF), among others [47]. However, it is crucial to note that supporting evidence for these approaches is weak, and the treatments are associated with high costs and significant heterogeneity. In contrast, GCs have gained attention as a relatively economical and widely available treatment for RIF. Exploring their effectiveness and usage patterns is considered a topic worthy of in-depth study. The main detailed information about clinical articles on the use of GCs (dexamethasone and prednisolone) for the treatment of RIF is presented in Table 1.

**Table 1:** Clinical research on the use of glucocorticoids and glucocorticoid combinations in the treatment of RIF.

	Author	Country	Study type	Intervention measures	Period	Age	Autoantibody	Primary outcome (clinical pregnancy rate)	Secondary outcome (live birth rate)
**Monotherapy**									
	Feng Zhang, 2017	China	Prospective randomized study	DXM intrauterine injection	2017.8.1	Exp:34.79% Con: NO	NA	Exp:87% Con: NO	Exp:38% Con: NO
	Sophie Cooper, 2019	UK	Cohort study	Prednisolone 10 mg orally	2010.6.22– 2017.5.22	Exp:35.9% Con:36%	NA		
	Zihua Wang, 2021	China	Prospective randomized study	Intrauterine infusion of dexamethasone sodium phosphate injection 5 mg (1 ml) before freeze-thawed embryo transfer	2018.6–2020. 10	Exp:33.24%(3.35) Con:33.29%(2.97)	NA	Exp:40% Con:22%	Exp:45% Con:27%
	Nana Ma, 2022	China, UK, Israel, Egypt	Meta analysis	Oral administration of cyclosporine A or prednisolone	2022.1.1	Exp:31.9% Con:32.3%	NA	NA	NA
	Yun Sun, 2023	China	Prospective randomized study	Prednisolone 10 mg orally	2018.8– 2021.8	NA	NA	Exp:82.7% Con:90.1%	Exp:37.8% Con:38.8%
**Combination therapy**									
	Jiao 2016	China	RCT	Start using prednisone (10 mg/day) and aspirin (100 mg/day) three months before the initiation of ovarian stimulation	NA	Exp: 31.10 ± 4.17 Con: 21.97 ± 3.44	ANA-positive	Exp: (53.3%) Con (30.1%)	NA
	Mitic 2019	Serbia	Prospective case-control study	Administer dexamethasone at 0.5 mg per day and ASA (Aspirin) at 100 mg per day. Dexamethasone treatment continues until the end of the 12th week of pregnancy, while ASA treatment continues until delivery	2017.9, 2018.10	Exp: 34.80 ± 4.06 Con: 36.29 ± 4.67	NA	Exp: (34.75%) Con: (28.77%)	NA
	Kolanska 2021	France	Cohort study	Combined treatment of prednisone 10 mg/day and LDA or intralipids.	2015.12, 2018.10	NA	NA	Exp: (43.64%) Con: (8.70%)	NA
	Zhou 2022	China	Retrospective cohort study	Treated with prednisone (10 mg/d) and aspirin (100 mg/d)	2017, 2020	Fresh: Exp:31.5 (29.0–35.0) Con:31.0 (29.0–35.0) Frozen: Exp: 30.0 (28.0–34.8) Con:30.0 (28.0–34.0)	All patients with positive thyroid autoimmune antibodies (TPOAb13 and/or TgAb14)	Fresh: Exp: (47/74) Con: (65/113) Frozen: Exp: (47/76) Con: (48/83)	Fresh: Exp: (35/74) Con: (56/113) Frozen: Exp: (34/76) Con: (39/83)
Glucocorticoid Combined with LMWH	Charalampos2013	Greece	Non-RCT	The dose of LMWH is 1 milligram per kilogram daily, and prednisolone is taken orally at 5 milligrams. If pregnant, prednisolone and heparin treatments will continue until the 12th week and the 34th week, respectively	2008.1, 2012.2	Exp:39 ± 3.0 Con:41.5 ± 3.8	Exp:(46.7%) Con:(21.4%)	Exp: (33.3%) Con: (14.3%)	NA
	Muhammad Fawzy2013	Egypt	RCT	Beginning, oral prednisolone 20 mg/day is administered. On the second day after oocyte retrieval, subcutaneous administration of LMWH is initiated at a dose of 1 mg/kg/day and continues until the conclusion of the pregnancy test	2008.1, 2012.10	Exp: 30.8 ± 4.9 Con: 31.3 ± 5.8	Exp: (40.7%) Con: (27.5%)	NA	NA
	Gavin Sacks 2022	Australia	RCT	Beginning, take 10 mg of prednisolone daily. Increase to 20 mg the day after egg retrieval or ovulation and initiate injections of 20 mg enoxaparin. Continue prednisolone until 12 weeks of pregnancy or a negative pregnancy test, and administer enoxaparin injections until 16 weeks of pregnancy or a negative pregnancy test	2016.2, 2020.4	Exp: 37.3 ± 4.5 Con: 36.8 ± 4.4	NA	Exp:(28%) Con:(6%)	NA
Combination Therapy with Glucocorticoid, Aspirin, LMWH, and IVIg	Xiao 2013	China	RCT	Dexamethasone is administered orally at a low dose of 5 mg three times daily, aspirin is given orally at a low dose of 50 mg once daily, and LMWH is administered subcutaneously at a daily dose of 5000 IU	2008.3, 2010.12	Exp: 27.92 ± 3.90; Con: 28.93 ± 4.63	APS	NA	Exp: (97.62%) Con: (83.91%)
Glucocorticoid Combined with Hydroxychloroquine	Gao 2021	China	RCT	The combined treatment with prednisone (10 mg/day) and HCQ (200 mg/day) starts on the third day of menstruation	2020.1, 2021.5	NA	ANA positive	Exp: (50.1%) Con: (20.0%)	NA
Glucocorticoid Combined with Antibiotics	Moffitt 1995	Swedish	RCT	16 mg oraI6-ex-methylprednis-olone for four evenings (treated with 250 mg oral tetracycline four times per day for 4 days).	1993.1, 1993.9	Exp:34.6 ± 3.8 Con:34.9 ± 4.0	NO	Exp:(32.14%) Con:(33.3%)	NA
	Zhang 2019	China	RCT	Treatment involves the use of intracervical dexamethasone in combination with antibiotics	2015.2, 2017.6	Exp 1: 33.45 ± 4.32 Exp2: 34.27 ± 3.61 Con:32.21 ± 4.16	CE	Exp1: (51.76%) Exp2: (25.00%) Con:(30.15%)	Exp1: 43.53%, Exp2: 12.50%, Con: 17.46%
	Ma 2023	China	RCT	Doxycycline (100 mg) administered twice daily for 14 days. During the same period, metronidazole (400 mg) is also administered twice daily	2019.7, 2021.6	Exp: 32.23 ± 3.41, Con: 33.05 ± 2.46	CE	Exp: (50%) Con:(30%)	Exp: (33.33%) Con: (45.46%)

### Pharmacological Mechanism of Glucocorticoids in RIF

GCs, named for their effects on glucose metabolism, are among the most common immunomodulators known for their anti-inflammatory and immunosuppressive properties. Overexpression of inflammatory factors in the endometrium is considered a major trigger for RIF [48]. uNK cells, crucial in uterine inflammation, express glucocorticoid, and estrogen receptors- beta (ER-β) receptors. Using GCs can decrease their abnormally high uNK cell numbers in women with RIF and modify their activity [11, 49]. This reduction in uNK cell cytotoxicity, including the release of inflammatory factors, creates a more favorable uterine environment for embryo implantation [50]. Studies have indicated that humans’ uNK cells can enhance and inhibit trophoblast invasion. In the initial stages of pregnancy, uNK cells control trophoblast invasion and contribute to changes in vascular functions that support placental growth. Nevertheless, numerous studies have observed the excessive accumulation of uNK cells in RIF. GCs also alleviate trophoblast growth and reduce trophoblast proliferation, which is inhibited by the excessive release of cytokines from abnormal uNK cells. Additionally, GCs can indirectly prevent further exacerbation of inflammation by preventing macrophages and immune effector cells from infiltrating the inflammatory site. This reduces the release of prostaglandins, reactive oxygen species, and cytokines such as IL-1 (Interleukin 1) and tumor necrosis factor-alpha (TNF-α), promoting the restoration of a normal endometrial state and healthy embryo implantation [51].

### The use of Dexamethasone in women with RIF

Dexamethasone, a synthetic, long-acting GCs with a duration of action between 36 and 54 h and a half-life of approximately 190 min, has multiple effects such as immunosuppressive, anti-apoptotic, anti-toxin, and anti-allergic effects [52]. It is widely used in treating various inflammatory and autoimmune disorders [53]. However, its placental permeability raises clinical concerns, as dexamethasone may disrupt fetal immune and endocrine development through transplacental transfer. Maternal exposure carries risks of gestational diabetes, hypertension, and increased susceptibility to infections due to immunosuppression [54, 55]. These safety considerations consequently lead to relatively few clinical trials using dexamethasone in the reproductive field, and its action on the endometrium is relatively limited [56].

As early as 1996, D. Bider et al. conducted a prospective and randomized study, finding that after oral administration of 0.5 mg dexamethasone daily for 5 days before and after a frozen embryo transfer in patients diagnosed with “pure” tubal infertility, the pregnancy rate in the dexamethasone-intervention group was not significantly improved [57]. Nevertheless, then few studies systematically focused on the use of dexamethasone in women with RIF. Despite its potential therapeutic relevance, few studies have systematically investigated the use of dexamethasone in women with RIF. Recent advances in the understanding of immune cell function, particularly the role of T cells in modulating inflammation and immune responses, have shifted attention to dexamethasone’s ability to regulate the Th1/Th2 cell ratio imbalance in the peripheral blood of RIF patients [51]. Additionally, dexamethasone has been shown to influence the ratio of uterine natural killer (uNK) cells [52], which may contribute to correcting immune dysregulation in RIF. These findings highlight the potential of dexamethasone as a therapeutic agent for addressing immune-related factors in RIF. The safety of dexamethasone in RIF was demonstrated by the fact that most patients had no pregnancy complications (87.5%), and nearly half delivered live fetuses (42.9%) by the end of the experiment. Subsequently, a trial in China conducted in 2021 included more patients (167) with RIF, following the intervention group received an intrauterine instillation of dexamethasone 5 mg one day before the transfer of frozen-thawed embryos. Researchers surprisedly found the clinical pregnancy rate in the dexamethasone group was significantly higher than that in the control group (40%/22%), while the rates of miscarriage, chemical pregnancies, and multiple pregnancies did not differ significantly [58]. Furthermore, a new study from the same center by Feng Zhang et al. confirmed the promising and effective treatment of Intrauterine perfusion of dexamethasone again, following the results that RIF patients are more likely to benefit than Recurrent Pregnancy Loss (RPL) patients [59].

In summary, intrauterine infusion and oral administration of dexamethasone represent distinct therapeutic modalities, each characterized by unique pharmacokinetic profiles, dosing regimens, and clinical implications [59]. While intrauterine infusion demonstrates potential advantages in specific clinical contexts, the current body of evidence is constrained by methodological limitations, including restricted sample sizes, heterogeneous patient populations across ethnic and geographic demographics, and substantial variability in treatment protocols. Notably, the existing literature lacks direct comparative studies between intrauterine and oral routes of dexamethasone administration, precluding definitive conclusions regarding their relative efficacy and safety profiles in the management of RIF. This evidentiary gap underscores the necessity for well-designed, prospective studies to elucidate the comparative therapeutic roles of these administration routes in clinical practice. Furthermore, the potential adverse effects associated with prolonged dexamethasone therapy warrant careful consideration. The pharmacological profile of dexamethasone is associated with a spectrum of systemic complications, including but not limited to metabolic disturbances (weight gain, diabetes mellitus), cardiovascular effects (hypertension), musculoskeletal complications (osteoporosis, muscular atrophy), and dermatological manifestations (skin thinning) [48]. While dexamethasone may offer therapeutic benefits in selected cases, the risk-benefit ratio necessitates thorough evaluation through rigorous clinical investigation to establish its optimal utilization parameters and safety profile in the context of RIF management.

### The use of prednisone and prednisolone in women with RIF

Prednisone and prednisolone, two common intermediate-acting GCs, have a duration of action between 12 and 36 h and a half-life of approximately 60 min [60]. Prednisone is rapidly converted to biologically active prednisolone by hepatic 11-beta hydroxysteroid dehydrogenase Type 1. Both have similar effects on the body, but prednisolone is considered the active form. Both are widely used clinically in a variety of immune-related diseases. Studies have found that they can maintain Treg cell function and increase the number of Treg cells [61]. In addition, prednisone can increase the proportion of Treg cells in decidual immune cells in vitro, inhibit the secretion of inflammatory cytokines, and reduce the number of Th17 cells [62]. Quenby et al. were the first to report the oral administration of prednisolone reduces pre-conceptual uNK cell levels in patients with RPL [63]. Over the past two decades, the use of prednisolone has extended beyond RPL to also include cases of RIF, becoming more widely used in clinical practice. Several studies revealed that in approximately half of RIF cases where the immune system is overactivated, prednisolone positively affects the immune profile of the endometrium. In 2019, Sophie Cooper et al. conducted a retrospective study in the UK, demonstrated that administering 10 mg of prednisolone per day up to 12 weeks of gestation to patients with recurrent reproductive failure (including RPL and RIF) and high uNK cells significantly reduced uNK cell concentrations (*P* < 0.001), but there was no significant difference in complications and pregnancy outcomes [64]. A previous UK-based randomized controlled trial (RCT) found no significant difference in live birth rates between the prednisolone and placebo groups [59], suggesting GCs may not be universally effective for RIF. This lack of efficacy could stem from patient heterogeneity, limited sample sizes, or insufficient endometrial drug concentrations. While Tang et al. reported higher live birth rates with corticosteroids in a specific subgroup of recurrent miscarriage (RM) patients with high uNK cells, these findings may not apply to the broader RIF population. Given these considerations, the conclusions presented in this review regarding the efficacy of GCs therapy in RIF patients may not be fully supported by the current body of evidence, underscoring the need for further research in this area. Yun Sun et al. recently conducted a large multicenter, randomized, double-blind, placebo-controlled study in China. Results showed that among patients with RIF, treatment with 10 mg prednisone did not improve live birth rate compared with placebo, however, the risk of biochemical miscarriage and preterm birth appeared to be increased [14].

Given these inconsistent findings, there is no consensus view on the utility of prednisolone in RIF which casts doubt on the treatment recommended by major guidelines. The prophylactic effect of prednisolone on RIF may be related to specific dosage, duration of intervention, and target population, which are not yet well-defined. Current trials have focused on doses ranging from 10 mg to 20 mg daily but have not aimed to compare the efficacy of different concentrations of prednisone/prednisolone with each other. Prednisolone is widely known to have the most significant side effect of complicating diabetes mellitus, but there was no significant effect in the trials mentioned above. The common complications reported in the trials were usually insomnia and a small number of gastrointestinal symptoms and headache manifestations. Further explorations on the optimal dose, timing, and population for prednisolone are needed to provide a more reliable basis for preventing miscarriage and revealing its potential side effects in clinical applications.

## Strategies for co-administration of glucocorticoids in RIF

While GCs may have a role in the treatment of RIF, current evidence supporting their efficacy and safety remains insufficient. GCs may not be necessary unless there are significant alterations in the immune system [65]. Recent trial results suggest that larger doses or longer durations of GCs might improve efficacy, but they also raise the risk of side effects. A comprehensive evaluation of the side effects of GCs use for RIF prevention is needed [32, 59]. Currently, GCs combination therapy for treating RIF primarily focuses on three directions: LDA, LMWH, and other related drugs. However, the co-administration of GCs in RIF requires a multifaceted approach that considers dosage, effectiveness, timing, patient selection, and monitoring [66]. These efforts will contribute to providing more effective treatment methods for RIF patients and improving their chances of successful fertility [17]. Continuous research and adherence to evidence-based practices are essential to optimize these strategies further.

### Glucocorticoids combined with LDA

Aspirin possesses properties that reduce blood viscosity and increase blood flow. This is due to its inhibition of cyclooxygenase-1 and reduction of thromboxane-2 production, leading to a shift from thromboxane A2 to prostacyclin I2, which promotes vasodilation, a necessary factor for embryo implantation [67]. LDA plays a crucial role in ART by improving blood flow to the uterus and ovaries, enhancing embryo implantation, and supporting early pregnancy. In most studies, the dose of aspirin is 100 mg, and it is also known to control ovarian hyperstimulation [68]. The daily use of LDA is considered safe as it does not affect the menstrual cycle, follicular phase, luteal phase length, or hormone levels throughout the menstrual cycle.

The combination of GCs and LDA is proposed to act synergistically, improving ovarian and endometrial blood flow while reducing local inflammation during the transfer process, thereby creating a more favorable environment for embryo implantation. Specifically, low-dose GCs such as prednisone suppress hyperactive immune responses, reduce inflammation, and enhance endometrial receptivity, providing a better immune environment for embryo implantation. Meanwhile, low-dose aspirin improves blood flow, increasing the blood supply to the ovaries and endometrium. The combined effect of these two drugs not only individually modulates immune responses and blood flow but may also enhance each other’s effects, optimizing both vascular supply and immune environment, thus creating more ideal conditions for embryo implantation [66]. However, the evidence on the effectiveness of this combination is mixed and still emerging. Previous studies have suggested that the combined treatment of prednisone and aspirin is effective for women with various autoimmune diseases [69]. Although most studies have not included populations without a history of IVF or RIF and did not report adverse complications, this suggests the potential benefits of GCs combined with LDA for RIF patients, which warrants further investigation to validate its effectiveness.

A study by Zhou et al. showed that adding aspirin to prednisone did not improve clinical pregnancy rates or miscarriage rates [66]. However, other researchers have used a combination therapy of levothyroxine, acetylsalicylic acid (ASA), and prednisolone (LT + ASA + P treatment). The results showed that women receiving LT + ASA + P treatment exhibited a higher ovarian response to gonadotropins, and pregnancy rates and implantation rates were significantly higher than in untreated ATA+ patients (PR/ET 25.6% and IR 17.7% compared to PR/ET 7.5% and IR 4.7%, respectively). The differential efficacy of whether to add levothyroxine to P + A combination therapy [70, 71], combined with relevant basic experiments, highlights the importance of thyroid hormones in women with thyroid dysfunction [72].

Based on this potential model, differentiating between different stages is crucial for developing effective treatment strategies. Therefore, further research is needed to explore whether levothyroxine should be included in P + A combination therapy for RIF caused by positive antithyroid antibodies and the timing of levothyroxine use.

For patients with autoantibodies undergoing IVF treatment, the adjunctive use of prednisone and aspirin is recommended, and several studies have confirmed its benefits. However, this combination therapy may lead to obstetric complications, particularly preterm birth and preeclampsia [73]. However, the study had a small sample size (only 11 patients) and focused on patients with anti-phospholipid antibodies who conceived naturally, with no history of IVF or RIF. Therefore, the extrapolation of its results may be limited. Due to inconsistencies in the initial treatment timing among different studies, the conclusions drawn also vary. For example, a study by Jiao et al. [74] found that starting prednisone (10 mg/day) and aspirin (100 mg/day) three months before ovulation induction resulted in significantly higher fertilization rates, pregnancy rates, and implantation rates in the combination therapy group compared to the untreated group. However, the miscarriage rate was also significantly higher in the combination therapy group than in the untreated group. On the other hand, a study by Hasegawa et al. initiated prednisone and aspirin two weeks before ovulation induction and showed that this combination therapy increased the pregnancy rate and reduced the miscarriage rate (pregnancy rate 40.6% vs. 14.8%, implantation rate 20.3% vs. 6.8%) [75]. Ando’s research involved the concurrent use of prednisone and aspirin from the first day of ovulation induction until clinical pregnancy, and it found that this combination therapy improved oocyte quality and pregnancy rates (pregnancy rate 38.03% vs. 7.35%) [76].

Similarly, for patients who are positive for anticoagulant lipid antibodies (ACA + ), those receiving methylprednisolone and aspirin treatment had significantly higher fertilization rates, pregnancy rates, and implantation rates compared to untreated ACA+ patients (fertilization rate 69.0% vs. 60.0%, pregnancy rate 46.4% vs. 33.3%, implantation rate 25.4% vs. 17.9%). However, two meta-analyses showed that aspirin alone had no significant benefits on clinical pregnancy or live birth rates [68]. This suggests that aspirin may play a different role in combination therapy, especially in RIF patients, where its effects remain uncertain. Previous studies have focused only on patients with ACA + , without specifically addressing RIF patients, thus further research is needed. In addition, there is a clinical trial from Serbia that investigates the efficacy of dexamethasone in combination with ASA. In this trial, dexamethasone was administered at a daily dose of 0.5 mg, and ASA was given at a daily dose of 100 mg. Dexamethasone treatment continued until the 12th week of pregnancy, while ASA treatment continued until delivery. The results showed that the implantation rate in the combination therapy group was higher than in the control group (26.53% vs. 15.92%), and this difference was statistically significant. Additionally, the clinical pregnancy rate for each starting cycle was also higher in the combination therapy group compared to the control group (43.59% vs. 28.92%), although the difference did not reach statistical significance. This demonstrates the potential benefits of dexamethasone in combination with ASA as adjunctive therapy for women undergoing IVF/ICSI (Intracytoplasmic sperm injection) procedures [77].

Overall, aspirin plays a crucial role in assisted reproductive therapy, but further in-depth research is needed to understand its effectiveness when used in combination with GCs, especially in different timing and dosing regimens. Additionally, exploring other adjunctive treatment methods, such as the efficacy of dexamethasone in combination with ASA, is also beneficial. Only through more clinical studies can we gain a more comprehensive understanding of the effectiveness and safety of these treatment approaches, providing better assisted reproductive therapy strategies for patients with infertility.

### Glucocorticoids in combination with LMWH

Among the proposed approaches to combat RIF, LMWH alone or in combination with GCs has been recommended abroad. The reason for using these two drugs is to improve the implantation and placental process. LMWH exerts its anticoagulant effect by inactivating factor Xa and promoting the action of antithrombin. Additionally, it increases the production of prolactin and insulin-like growth factor-1 (IGF-1), inhibits the production of insulin-like growth factor-binding protein-1 (IGFBP1), and regulates heparin-binding epidermal growth factor. Therefore, LMWH appears to have a positive role in various stages of embryo implantation, from facilitating the decidualization process to aiding in the attachment, differentiation, invasion of blastocysts into the endometrium, and early embryonic growth. All the evidence from basic research demonstrated that LMWH and prednisolone have, at least in some trials, efficacy in improving implantation. In actuality, data regarding the combined use of LMWH and prednisone in clinics are currently limited.

In 2013, Charalampos Siristatidis et al. conducted a non-randomized clinical trial that included 52 participants to investigate the effect of the coadministration of LMWH and prednisolone [78]. Presented results revealed the clinical pregnancy rates and crude live birth rates were improved by more than tripled. However, the same hospital conducted a new study involving 105 participants in 2018, which included examinations of laboratory and clinical parameters, as well as maternal side effects, showed no significant differences in pregnancy rates and miscarriage rates between the group receiving combined LMWH and prednisolone and the group not using adjuvant medications [79]. Increasing the sample size appeared to have a significant impact on the results. A prospective quasi-randomized controlled study from Egypt involving more patients (295 participants), following LMWH 1 mg/kg/day combined prednisolone (20 mg/day). They found significant differences in pregnancy rate (23.9% vs. 14.7%) and implantation rate (38.6% vs. 24.6%) in the combined therapy group compared to the control group [80]. Enoxaparin is a type of LMWH widely used in Australia. It is commonly employed for the prevention and treatment of conditions such as deep vein thrombosis, pulmonary embolism, and prophylactic anticoagulation after cardiopulmonary resuscitation. Studies have shown that women treated with the Bondi (prednisolone + enoxaparin) protocol have a significantly higher live birth rate compared to the normal protocol, which may be attributed to its regulatory effect on elevated NK cell activity. Enoxaparin combined with prednisolone for the treatment of RIF is a potentially effective treatment option in clinical practice.

Although preliminary findings from pilot studies suggest a trend towards improved pregnancy outcomes with the combined use of LMWH and prednisone, the actual effectiveness of this combination therapy cannot be confirmed at present due to small sample sizes. Noting that these results were obtained without blinding, or randomization, and may have been affected by unknown confounding factors and selection bias. Therefore, further validation of the benefits of combined therapy in large multicenter RCTs is still necessary, especially focusing on improving clinical pregnancy rates and live birth rates in the treatment of RIF. This approach may offer new avenues to improve patients’ fertility success rates [79].

### The combined therapy of glucocorticoids, LDA, LMWH, and Intravenous Immunoglobulin

The combination of prednisone, aspirin, LMWH, and intravenous immunoglobulin (IVIg) is a common treatment for RIF induced by APS. IVIg can neutralize pathogenic autoantibodies by interacting with Fc or Fab receptors through the Fc portion of immunoglobulins or by passively acting as specific antibodies [81]. In addition, IVIg can block the binding of autoantibodies at the level of endothelial cells, thereby eliminating the prothrombotic effect of antiphospholipid antibodies and endothelial cell activation [82]. IVIg can also regulate the production of pro-inflammatory and anti-inflammatory cytokines and interact with complement activation. However, IVIg therapy is expensive, limiting its widespread clinical use, and its effectiveness still needs further verification due to the lack of large-scale clinical trials.

Xiao’s study was the first to evaluate the effectiveness of combined therapy with prednisone, aspirin, LMWH, and IVIg in APS patients [83]. In this trial, prednisone was administered at a low dose of 5 mg three times a day, aspirin at a low dose of 50 mg once daily, and LMWH at a daily dose of 5000 IU by subcutaneous injection. The results showed that patients receiving the combination therapy had a significantly higher live birth rate compared to patients receiving traditional treatment with only prednisone and aspirin (97.62% vs. 91.73%). Additionally, the rate of obstetric complications was significantly reduced in the comprehensive treatment group. Contrary to these results, The study by Kiper Aslan et al. focused on luteal phase combination therapy and did not observe an improvement in live birth rates, possibly due to the absence of IVIG and the use of different doses (4000 IU LMWH, 150 mg aspirin, and 16 mg prednisolone) [84]. Additionally, there were significant differences in the study populations: Xiao’s study included patients with natural conceptions and APS, while Aslan’s study targeted patients with IVF failure and no evidence of immunological abnormalities. These differences may account for the inconsistent results. Therefore, the efficacy of combination therapy in the more specific population of RIF warrants further investigation.

Undoubtedly, significant heterogeneity exists among studies, even in the definition used for RIF patients. The mentioned studies were not blinded and had limited stringency in their inclusion criteria. Large-scale RCTs are needed in the future to confirm the efficacy and side effects of comprehensive treatment in APS-induced RIF. Besides, the trend of comprehensive treatment in improving live birth rates and reducing obstetric morbidity rates is still worth further investigation.

### Glucocorticoids combined with progesterone

Progesterone, a sex steroid hormone, is crucial for maintaining pregnancy. It functions by regulating the maternal immune system, inducing progesterone-induced blocking factor to suppress the activity of NK cells, Th2-dominant cytokines, and the production of galectin-1 by maternal immune cells. A systematic review and meta-analysis conducted by Saccone et al. demonstrated that the early use of progesterone in women with recurrent miscarriages offers significant benefits, suggesting that progesterone use may help prevent early pregnancy risks [85]. Moreover, a study by Lédée et al. in 2016 focused on women with unexplained RIF and employed a combination treatment approach, including high-dose progesterone (1200 mg/day) and prednisolone (20 mg/day) [86]. Progesterone administration began on the day of egg retrieval and continued for 8 weeks after embryo transfer, while prednisolone supplementation started on the third day of ovarian stimulation and was complemented with vitamin E. The results revealed that women receiving this combination treatment achieved a live birth rate of 50%. Immunological analyses also reported a decrease in the Interleukin 18 (IL-18)/ tumor necrosis factor-like weak inducer of apoptosis (TWEAK) mRNA ratio and Interleukin 15 (IL-15)/ fibroblast growth factor-inducible (Fn-14) mRNA ratio, further indicating the positive impact of this treatment regimen on improving pregnancy outcomes [86]. With the advent of algorithms such as virtual screening and molecular dynamics simulations, Soodeh Mahdian and colleagues first investigated the potential role of prednisone and progesterone in suppressing TNF-α in patients with RIF [87]. Prednisone and progesterone tend to bind well to TNF-α. In this case, inhibition of TNF-α may be effective in the treatment of patients with RIF.

In general, the combination of prednisone and progesterone shows potential benefits in addressing embryo implantation failure following IVF. However, thorough clinical trials and in-depth studies on the mechanism of action are essential to fully validate the effectiveness and safety of this combined therapy. Such research endeavors will contribute to the development of more efficient assisted reproductive strategies aimed at enhancing women’s fertility.

### Glucocorticoids combined with Hydroxychloroquine (HCQ)

HCQ is considered a safe immunosuppressant for pregnant women. Specifically, HCQ can down-regulate Th17-related cytokines and functions and upregulate Treg-related cytokines and functions [88]. It is recommended for use throughout pregnancy in cases of SLE due to its immunomodulatory and anti-inflammatory properties [89]. Meng et al. conducted a retrospective analysis of patients who experienced IVF-ET failure and had an elevated Th1/Th2 ratio. They found that although the treatment group did not show statistically significant differences in key indicators such as live birth rate and clinical pregnancy rate, the overall trend suggested that the treatment may have a positive effect on improving pregnancy outcomes [90]. Building on this, Gao et al. further explored a treatment regimen combining prednisone (10 mg/day) with hydroxychloroquine (200 mg/day), initiated on the third day of menstruation. They discovered that this regimen could significantly enhance implantation rates, biochemical pregnancy rates, and clinical pregnancy rates in immunopositive patients, while also reducing the rate of pregnancy loss [91]. Furthermore, Lian et al. revealed a significant improvement in fertilization rate (*P* = 0.017), implantation rate(*P* = 0.032), and clinical pregnancy rate (*P* = 0.028) in the prednisone combined HCQ group than the alone prednisone group [92]. However, these were retrospective studies with a limited sample size, and the clinical applicability of the conclusions is thus limited. To gain a deeper understanding of the value of prednisone combined with the HCQ treatment regimen in fresh embryo transfer and frozen embryo transfer cycles for patients with RIF, more prospective research and large-scale RCTs are needed.

### Glucocorticoids in combination with antibiotics

Chronic endometritis (CE) is a persistent inflammation of the endometrium, and oral antibiotics are the main route of administration for the treatment of CE. They increase the receptivity of the endometrium by killing bacteria, thus improving implantation during pregnancy.

A study by Nana Ma et al. in mares found that combining oral doxycycline with intrauterine infusions of gentamicin and dexamethasone significantly improved outcomes compared to oral antibiotics alone, with higher implantation rates (30.95% vs. 26.67%), clinical pregnancy rates (50% vs. 30%), ongoing pregnancy rates (45.45% vs. 33.33%), and live birth rates (45.45% vs. 33.33%) [93]. Zhang et al. reported that 77.98% of patients achieved EC remission using intrauterine infusions of dexamethasone with gentamicin or clindamycin, and the combination therapy group had higher implantation and clinical pregnancy rates compared to controls [94]. However, other studies have shown conflicting results; for instance, a study involving patients with RIF found no significant differences in outcomes between groups receiving different antibiotic treatments.

Studies indicate that combining GCs with antibiotics improves reproductive outcomes, although some conflicting results exist. Further research is needed to assess endometrial receptivity and the local immune state in each RIF patient to validate the role of dexamethasone in regulating the immune environment and improving endometrial receptivity. Future research should focus on personalized treatment approaches, assessing endometrial receptivity, and conducting high-quality randomized clinical trials to determine the most effective regimens. Additionally, developing novel drug delivery systems, such as intrauterine sustained release systems and biodegradable nanocarriers, could enhance treatment efficacy and bioavailability.

### Glucocorticoids in combination with intralipid

Intralipid, an intravenous fat emulsion containing 20 g of soyabean oil, 1.2 g of phospholipids, 2.25 g of glycerin, and water for injection, has demonstrated potential immunomodulatory effects in reproductive medicine, particularly in reducing NK cell activity and enhancing endometrial receptivity [95]. Although its overall mechanistic effect on the immune system remains unclear, when combined with GCs, this therapy aims to synergistically modulate the immune environment, possibly mediated through short fatty acids stimulating PPARγ receptors, thereby improving implantation outcomes. Research by Canella et al. [96]. suggested that Intralipid can influence the Th1/Th2 balance, shifting it toward a Th2-dominant response, which is more conducive to successful implantation. Both Intralipid and GCs can suppress NK cell cytotoxicity, potentially leading to a more receptive endometrial environment. What’s more, a multicenter cohort study [97] assessed the impact of immunomodulatory therapy, incorporating corticosteroids and intravenous Intralipid infusions, on live birth rates in women with RIF undergoing assisted reproduction. The results indicated that the treatment group exhibited a significantly higher live birth rate compared to the control group (20.9% vs. 15.8%, OR 1.4, 95% CI 1.29–1.53, *p* < 0.001). A RCT of 105 RIF subjects found that clinical pregnancy in women who received intralipid vs placebo was 3.1 times (1.02–9.70 95% CI, *P* = 0.046) [98]. As such, intralipids are an effective and safe therapy in women with a history of RIF, while the best way forward may be to compare immunological testing between women who achieve successful live births after receiving Intralipid and those who do not. Until the intricacies of endometrial receptivity are fully understood, the need for larger, better-powered randomized controlled studies cannot be overstated.

### Glucocorticoids in combination with TNF-α Inhibitors

Winger EE et al. [99] identified elevated levels of tumor necrosis factor-alpha (TNF-α), a pro-inflammatory cytokine, as a contributing factor in immune-mediated implantation failure. Furthermore, TNF-α inhibitors, such as infliximab and adalimumab, have been proposed as potential treatments to modulate excessive inflammatory responses in RIF. TNF-α inhibitors reduce pro-inflammatory cytokine levels, potentially creating an immune profile conducive to implantation. When used in combination with GCs, the immunomodulatory effects of TNF-α inhibitors may be enhanced. Cortisol alleviates systemic inflammation by inhibiting T cell activation and reducing cytokine secretion, thereby optimizing immune tolerance. On the other hand, TNF-α inhibitors specifically target TNF-α, suppressing its role in both local and systemic inflammation. The combined use of these two agents improves immune responses through a synergistic effect: cortisol suppresses excessive activation of the immune system, increases T regulatory cells, and promotes immune tolerance; TNF-α inhibitors reduce the activity of Th1 cells and promote the balance of Th2 cells, thus maintaining immune homeostasis. The combination not only improves systemic inflammation but also optimizes the immune environment of the endometrium, which is conducive to embryo implantation [99]. Furthermore, TNF-α inhibitors may promote follicular development and egg quality by reducing TNF-α levels in the follicular fluid, while cortisol supports reproductive function by regulating immune tolerance and maintaining endocrine balance. In conclusion, the synergistic effect of TNF-α inhibitors and cortisol, through multiple immunomodulatory mechanisms, improves endometrial receptivity and supports successful pregnancy [100, 101]. However, a systematic review and meta-analysis [101], which evaluated the efficacy of TNF-α inhibitors and GCs in improving reproductive outcomes, concluded that current evidence is insufficient to support the routine use of these immunotherapies for enhancing live birth rates in women undergoing IVF or preventing idiopathic recurrent pregnancy loss.

## Conclusion

RIF is a challenging infertility condition with no unified diagnostic and treatment standards. The immunology of RIF is complex and not fully understood, with immune cells like NK cells and T cells playing crucial roles and representing potential therapeutic targets. GCs, commonly used as immunomodulators, can suppress endometrial inflammation, reduce uterine cytotoxic cell activity, and improve the implantation environment. However, evidence of their efficacy and safety in treating RIF is inconsistent. Different types, dosages, routes, and timings of GCs may have varying effects and side effects, necessitating personalized treatment regimens. Therefore, caution is advised in using GCs to prevent RIF. More large-scale, multicenter, RCTs are needed to verify their effectiveness, elucidate mechanisms of action, and account for individual variations. Current research on combination therapy with GCs has primarily focused on their use with LDA and LMWH. Given the complexity of clinical practice and variations in RIF cases, further high-quality research is required to determine appropriate dosages, durations, and administration methods, with efficacy and risk assessments demonstrated in well-designed, adequately powered trials.
